# Akt expression and compartmentalization in prediction of clinical outcome in HER2-positive metastatic breast cancer patients treated with trastuzumab

**DOI:** 10.3892/ijo.2012.1576

**Published:** 2012-07-27

**Authors:** PETER GRELL, PAVEL FABIAN, MARTA KHOYLOU, LENKA RADOVA, ONDREJ SLABY, ROMAN HRSTKA, ROSTISLAV VYZULA, MARIAN HAJDUCH, MAREK SVOBODA

**Affiliations:** 1 Departments of Comprehensive Cancer Care and; 2 Pathology, Masaryk Memorial Cancer Institute, 656 53 Brno;; 3 Institute of Molecular and Translational Medicine, Faculty of Medicine and Dentistry, Palacky University, 775 15 Olomouc, Czech Republic

**Keywords:** breast cancer, HER2 protein, trastuzumab, AKT1 protein, AKT2 protein, prognosis

## Abstract

Trastuzumab is effective in about half of HER2-positive breast cancer patients. The PI3K/Akt signalling pathway plays an important role in the process of primary and secondary resistance to anti-HER2 targeted therapy. We evaluated the relationship between expression, activation and subcellular localization of selected Akt isoforms and response to trastuzumab-based anti-HER2 targeted therapy in patients with HER2-positive metastatic breast cancer. Seventy-four women with verified HER2-positive breast cancer were treated with trastuzumab for metastatic disease. Immunohistochemistry was used to evaluate Akt1, Akt2, pAkt Thr308 and pAkt Ser473 expression. For pAkt, cytoplasmic and nuclear fractions were assessed separately. Even though Akt isoforms were expressed in the majority of tumours, activated Akt (pAkt) was present in the cytoplasm only and not in the nucleus in >20% of tumours, and there was no pAkt at all in another 7–13% of tumours. Patients whose tumours showed strong Akt2 expression and had pAkt (pAkt-Thr308 and/or pAkt-Ser473) detectable in the cytoplasm as well as nucleus (n+c), exhibited improved time to progression (TTP) and overall survival from the initiation of trastuzumab therapy (OSt). Patients with tumours with strong Akt2 and pAkt Thr308 (n+c) had superior TTP (17.0 vs. 7.6 months, P=0.024; HR 0.52) and OSt (51.8 vs. 16.8 months, P=0.0009; HR 0.34) compared to other tumours. Similar results were found for strong Akt2 and pAkt Ser473 (n+c): TTP 13.1 vs. 7.2 months (P=0.085, HR 0.62) and OSt 50.8 vs. 17.0 months (P=0.009; HR 0.45). This study is the first to prove the significance of Akt kinase isoform, activity and compartmentalization for the prediction of response to trastuzumab-based therapy in patients with HER2-positive metastatic breast cancer.

## Introduction

Breast cancer is the most common cancer in women in western countries (following skin neoplasms). Advances in gene profiling and molecular methods have brought about a shift from traditional morphology-based diagnostics to more precise molecular classification of breast cancers. Specific gene profiling and protein expressions are reflected in a distinct disease behaviour, prognosis and treatment response for each subgroup of breast cancers. HER2-positive breast cancer is one of these subgroups and is characterized by overexpression of HER2 protein and/or amplification of HER2/neu gene. The HER2/neu gene (also known as ErbB-2) is a proto-oncogene and encodes the trans-membrane receptor protein HER2. HER2 is a member of the human epidermal growth factor receptor family and is overexpressed/amplified in ∼15–25% of breast cancers (1,2). To reflect the high importance of HER2 receptor signalling pathway for HER2-positive breast cancers, molecular taxonomy refers to HER2-positive breast cancers as a separate entity. HER2 receptor activation, either by homodimerization when there is an abundance of HER2 receptors or heterodimerization after stimulation of certain dimerization partner receptors with a pertinent ligand, leads to subsequent activation of PI3K/Akt and Ras/ERK/MAPK signalling pathways responsible for the naturally high proliferation and aggressiveness of these tumours. Without targeted anti-HER2 therapy, currently available in the form of monoclonal antibodies and tyrosin kinase inhibitors, HER2-positive breast cancer would continue to have one of the poorest prognosis among breast cancers.

Despite the efficacy of targeted therapy, about half of HER2-positive breast cancer patients have primary resistance to trastuzumab or acquire resistance in the course of trastuzumab therapy. Disease progression at the first radiological assessment, or primary resistance, was observed in 50% of patient with metastatic disease treated with single-agent trastuzumab (3–5). Similar results were obtained for combined treatment with trastuzumab and chemotherapy in metastatic disease (50% overall response rate/ORR/) or in adjuvant settings (disease-free survival/DFS/odds ratio/OR/ = 0.69 and locoregional recurrence OR = 0.53) (6,7).

Potential mechanisms of resistance to trastuzumab include factors related to HER2 interactions with other members of the HER family or trastuzumab, including the loss of, or increased, HER2 expression; increased HER1 or HER3 expression; increased TGF-α expres sion (a ligand for EGFR/HER1); steric hindrance of HER2-antibody interaction by membrane-associated glycoproteins; and inhibition of trastuzumab binding by HER2 ECD (extracellular domain) fragments cleaved from the HER2 receptor. Incomplete HER family blockade might be an important resistance mechanism, since it could allow another HER receptor to compensate when one receptor is blocked (8). Resistance to trastuzumab might also arise through alternative signalling pathways or through constitutive activation of the PI3K/Akt signalling pathway, which is activated by HER2 signalling (and therefore suppressed by HER2 inhibitors such as trastuzumab). Constitutive activation might occur, for example, due to mutations in the PIK3CA gene and/or loss of PTEN. Similar to HER2, the IGF-1R, which can form heterodimers or heterotrimers with HER2, activates the PI3K/Akt pathway and this mechanism is thought to be an important source of trastuzumab resistance. Conversely, PTEN suppresses the activation of the PI3K/Akt pathway and loss of PTEN activity results in increased Akt activity and resistance to trastuzumab (8). Hence, PI3K/Akt pathway and Akt kinase seem to play a crucial role in oncogenic potential of HER2 and the development of resistance to anti-HER2 targeted therapy (8–10).

Activation (phosphorylation) of Akt is a multistep process (11). The signalling cascade is initiated at cell membrane when a ligand binds to a receptor (such as growth factor receptors, integrins, etc.) and triggers conformational change of the receptor. Consequently, this attracts PI3K (phosphatidylinositol 3-kinases) to the plasma membrane and activates it. Phosphorylated PI3K generates the second messenger PIP3 that recruits Akt to the inner cell membrane, enabling phosphorylation of the Akt by its activating kinases, at the threonine 308 residue (by PDK1) and at the serine 473 residue [by rictor-mTOR complex in response to growth factor stimulation (12) or by DNA-PK in response to DNA damage (13)]. Once Akt is activated, it dissociates from the plasma membrane and proceeds to phosphorylate both cytoplasmic and nuclear downstream effectors.

Akt family consists of three serine/threonine kinases (Akt1, Akt2 and Akt3), with slightly different preferences in their downstream effectors and varied presence in different types of tissue. HER2-positive breast cancer cell lines and primary tumours were found to have high expressions of Akt1, Akt2 and their activated forms (14–18). Various Akt isoforms were also found to have different biological functions in breast cancer. Akt1 accelerates tumourigenesis through increased cell proliferation and is thus predominantly involved in the control of cell malignant transformation, whereas, simultaneously, Akt1 suppresses tumour invasion (19). Akt2 overexpression enhances invasive potential of breast cancer cells and their ability to metastasize, Akt2 expression and activity suppress anoikis and apoptosis caused by deprivation of nutrients (19,20). The role of Akt3 in breast cancer tumourigenesis is less clear. A number of studies examined the role of Akt, especially the Akt1 and Akt2, in breast cancer and its prognostic and predictive value (16–18,21–28). However, no study so far have evaluated the correlation between total Akt expression, subcellular localization of the activated phosphorylated Akt (pAkt) and the results of anti-HER2 targeted anticancer therapy that, through HER2 receptor, significantly affects this signalling pathway. Therefore, we sought to explore the relationship between activation and compartmentalization of Akt1 and Akt2 and the outcome of targeted therapy in a sample of patients with metastatic HER2-positive breast cancer treated with trastuzumab.

## Materials and methods

We enrolled 74 breast cancer patients treated between 2001 and 2009 at the Masaryk Memorial Cancer Institute (Brno, Czech Republic) for metastatic disease. The eligibility criteria included: confirmed HER2 positivity, availability of formalin-fixed and paraffin-embedded (FFPE) tissue samples of primary tumours for immunohistochemistry evaluation, history of trastuzumab based therapy for metastatic breast cancer and availability of medical records for review. Informed consent was obtained from each participating subject. Clinical data were reviewed retrospectively from medical records. Immunohistochemistry (IHC) evaluation was performed on tissue microarrays (TMAs). TMAs were constructed from FFPE tissue sections using a technique developed at our institution. The expression of HER2 protein was determined by Dako Herceptest (Dako, Sweden) and scored on a qualitative scale from 0 to 3+ according to Dako manual and American Society of Clinical Oncology/College of American Pathologists guideline recommendations for human epidermal growth factor receptor 2 testing in breast cancer. HER2 gene status was evaluated by FISH method using Abbott PathVysion HER2 kit (Abbott Laboratories, USA). HER2 gene status was considered as positive (FISH amplified) in case where a HER2 gene/centromer of chromosome 17 ratio was higher than 2.2 or if the number of HER2 gene copies was higher than 6 per nucleus as measured by FISH. All tumours were IHC 3+ and/or FISH-positive. The estrogen receptor (ER) and progesterone receptor (PgR) status was examined by IHC, using antibodies provided by Lab Vision (SP1 resp. SP2 monoclonal rabbit antibody, Lab Vision Thermo Fisher Scientific, Fremont, CA, USA). ER and PgR status was considered positive if >1% of cells were stained in cell nuclei, and was considered negative in all other cases. Similarly, Akt expression was assessed using IHC. Murine monoclonal antibody was used for Akt1 IHC and rabbit monoclonal antibody was used for Akt2 expression detection, both purchased from Cell Signalling Technology (Beverly, MA, USA) and applied according to the manual provided by the manufacturer. The tumours with <5% of cells stained for protein were considered Akt1/Akt2-negative. If ≥5% cells were stained with the respective antibodies, the tumours were considered Akt1 or Akt2-positive. The total Akt1 or Akt2 expression was considered to be strong if >80% of cells were stained positive for the respective antibody. Rabbit monoclonal antibodies, both from Cell Signalling Technology, were used to detect phosphorylated (activated) Akt (pAkt) at Thr308 and phosphorylated Akt at Ser473. The expression was considered positive if ≥5% tumour cells were stained with the antibody. Cytoplasmic (c) and nuclear (n) fractions were assessed separately for pAkt expression and tumours were divided into three groups: group 1, pAkt expression negative; group 2, cytoplasmic-only pAkt expression (pAkt-c); group 3, nuclear and cytoplasmic pAkt expression (pAkt-n+c). The immunohistochemistry assessment was performed by pathologists with an extensive experience in evaluating tissue arrays and blinded to patient characteristic and treatment outcomes.

### Statistical analysis

Data are summarized using standard descriptive statistics and frequency tabulations. Correlations between expressions of various biomarkers (Akt1, Akt2, pAkt Thr308, pAkt Ser473, ER, PgR) were analyzed using Spearman’s rank correlation test. χ^2^ tests were used to determine correlation between Akt expression or activation status and other clinical or pathological characteristics. Response to therapy was evaluated with RECIST criteria version 1.1. Time to progression (TTP) was defined as the time from trastuzumab-based treatment initiation to the first documented objective disease progression. Overall survival was defined as the time from trastuzumab-based therapy initiation to death from any cause (OSt), or time from diagnosis of a metastatic breast cancer to death from any cause (OSm). Survival data were plotted using the Kaplan-Meier method. The log-rank test was used to analyze differences in TTP and OS. Univariate and multivariate analyses of predictive factors were performed using Cox’s proportional hazard regression. All tests were two-sided and the significance level was set at α = 0.05. Statistical analysis was performed with the support of MedCalc 9.1 software.

## Results

### Patient and tumour characteristics

We analyzed data from 74 patients. Patient and tumour characteristics are summarized in Table I. All patients were female. Sixty-five patients were diagnosed with early breast cancer and progressed to metastatic cancer, 9 patients were diagnosed with metastatic breast cancer. All patients were treated with trastuzumab-based therapy only for metastatic breast cancer. The use of trastuzumab corresponded to the knowledge then available. Three patients were treated with trastuzumab monotherapy, all other patients (96%) were treated with a combination of chemotherapy and trastuzumab. Most frequently, trastuzumab was combined with taxanes (57 patients, 77%) and was administered as the first line therapy for metastatic breast cancer (44 patients, 59.5%). Initial trastuzumab therapy led to complete remission in 9 patients (12.2%) and partial remissions in 33 patients (44.6%). Overall response rate was 56.8%. Stable disease as the best response was achieved in 19 patients (25.6%) and no response to therapy with disease progression was seen in 8 patients (10.8%). Clinical benefit (CR or PR or SD for at least 6 months) was achieved in 55 patients (74.3%). Response could not be clearly identified in 5 patients, in whom the disease metastasized predominantly to the skeleton. Median follow-up time was 41 months. Disease progression was documented in 64 (86.5%) patients. Median TTP for the entire group was 9.2 months (range from 1.3 to 56.2 months), median OSt was 20.1 months (range 1.3 to 68.3 months) and OSm was 29.8 months (range from 1.75 to 83 months).

The majority of tumours were invasive ductal carcinomas (59 patients, 79.7%) and scored as grade 3 (59.5%), 32 patients had an ER-positive tumour (43.2%), 20 patients had a PgR-positive tumour (27.0%) and 35 had an ER and/or PgR-positive tumour (47.3%).

### Akt expression and compartmentalization in breast cancer

**Figure 1:**
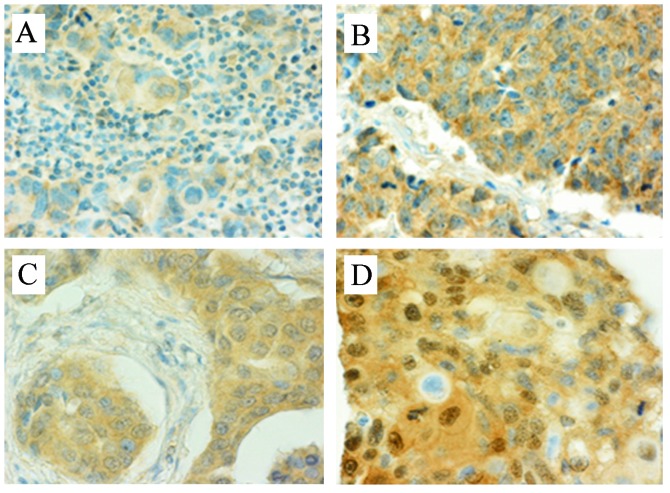
Examples of IHC assessment of Akt expression and compartmentalization.

Seventy-four primary tumour samples were analyzed for Akt1, Akt2, pAkt Thr308 and pAkt Ser408 expression and scored as described above. Seventeen tumours (23.0%) were negative on Akt1 staining, 46 (62.1%) were Akt1 weak positive and 9 (12.2%) were strong positive, results for two tumours were not interpretable. There were no Akt2-negative tumours, 45 (60.8%) samples were weak positive (Fig. 1A) and 26 (35.1%) were strong positive on Akt2 staining (Fig. 1B), results for three tumours were not interpretable. For phosphorylated Akt, cytoplasmic and nuclear fractions were assessed separately. Ten tumours (13.5%) were negative on any pAkt Thr308 staining, 22 tumours (29.7%) were positive on cytoplasmic staining only (Fig. 1C) and 38 (51.4%) were positive on both nuclear and cytoplasmic (n+c) staining (Fig. 1D), 4 cases (5.4%) could not be assessed. For pAkt Ser473 staining, 5 cases were negative, 16 cases (21.6%) were positive on cytoplasmic staining only and 49 (66.2%) were positive on both nuclear and cytoplasmic staining, 4 cases (5.4%) could not be interpreted. Akt expression results are summarized in Table II.

We found no correlation between expression of Akt1, Akt2 or activated forms of Akt and expression of estrogen or progesterone receptors or tumour grading.

### Correlation of Akt expression and compartmentalization with time to progression

For patients with metastatic breast cancer treated with trastuzumab, we tested the predictive value of expression of distinct forms of Akt. There was no association between total Akt1 expression and TTP. We observed an unexpected trend towards improved TTP in patients with tumours with strong Akt2 expression (13.1 vs. 7.2 months) but the difference was not statistically significant (P=0.133; HR 0.682; 95% CI 0.42–1.12). We evaluated cytoplasmic and nuclear localization of pAkt separately. Even though we did not observe any statistically significant difference in TTP with respect to different pAkt Thr308 or pAkt Ser473 localization, there was, once again, an unexpected trend to longer TTP in patients with activated Akt in both the cytoplasm and nucleus (pAkt-n+c) compared to tumours with pAkt detected in the cytoplasm only (pAkt-c): pAkt Thr308-n+c vs. pAkt Thr308-c, 10.2 vs. 8.3 months; pAkt Ser473-n+c vs. pAkt Ser473-c, 9.4 vs. 8.1 months, none of these results were, however, statistically significant.

**Figure 2:**
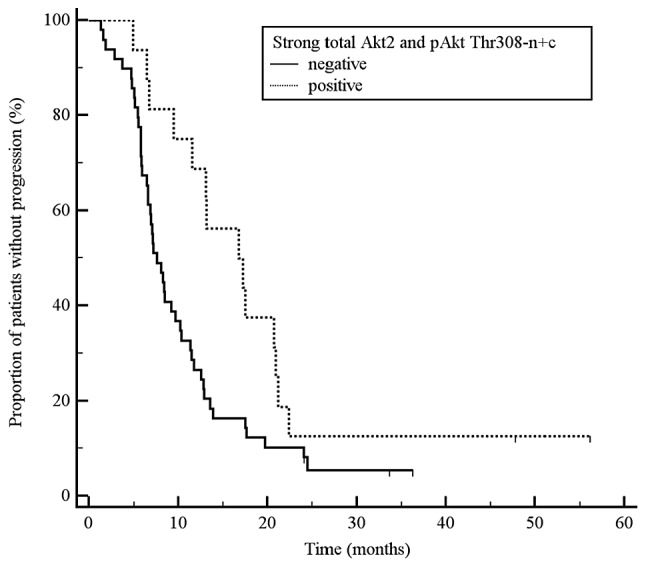
Kaplan-Meier plots of time to progression for Akt2 expression rate and concurrent compartmentalization of activated pAkt Thr308.

**Figure 3:**
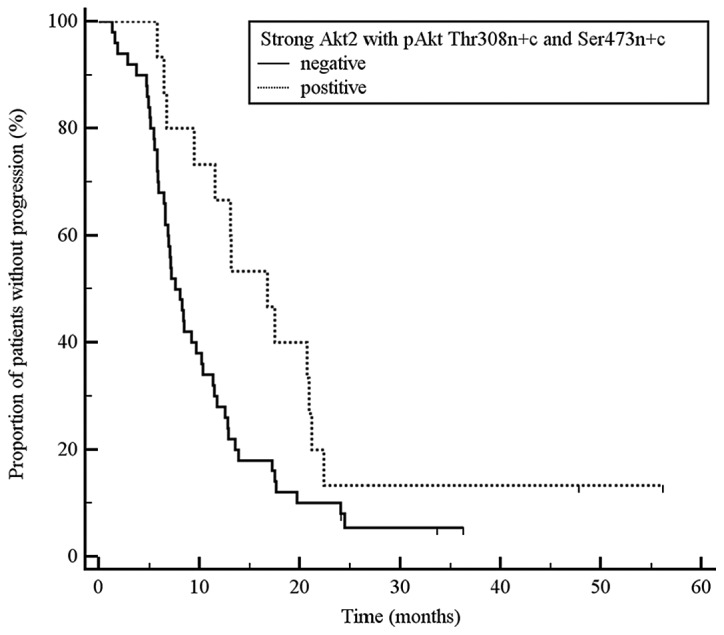
Kaplan-Meier plots of time to progression for the Akt2 expression rate and concurrent compartmentalization of both activated pAkt Thr308 and pAkt Ser473.

There was an interesting trend to a longer time to progression in patients whose tumours had strong expression of total Akt2 or activated Akt (pAkt) detectable in both the cytoplasm and nucleus. Consequently, we explored interrelationships between these factors and found that patients with strong Akt2 expression and activated Akt (pAkt) detectable in both the cytoplasm and nucleus had statistically significantly longer time to progression than other patients (any = any total Akt2 expression and simultaneously pAkt limited to cytoplasm only). The results were as follows: a) tumours with strong total Akt2 expression and simultaneous pAkt Thr308 n+c vs. all other tumours: TTP 17.0 vs. 7.6 months, P=0.024, HR 0.52, 95% CI 0.31–0.87; Fig. 2; b) tumours with strong total Akt2 expression and simultaneous pAkt Ser473 n+c vs. all other tumours: TTP 13.1 vs. 7.2 months, P=0.085, HR 0.62, 95% CI 0.37–1.03; c) tumours with strong total Akt2 expression and simultaneous pAkt Ser473 n+c and Thr308 n+c vs. all other tumours: 16.8 vs. 7.6 months, P=0.029, HR 0.52, 95% CI 0.30–0.88; Fig. 3.

We did not identify any correlation between survival (TTP or OSt) and Akt1 expression and its combination with various pAkt localizations.

### Correlation of Akt expression and compartmentalization with response to therapy

**Figure 4:**
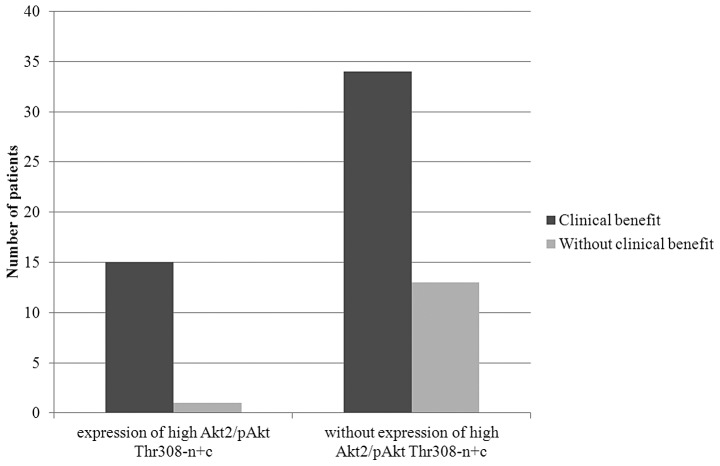
Correlation between clinical benefit and Akt kinase expression.

We found no statistically significant correlations between expression of Akt isoforms or Akt compartmentalization and trastuzumab therapy overall response rate or clinical benefit rate. Patients with concurrent strong total Akt2 expression and pAkt presence in both the cytoplasm and nucleus were more likely to achieve clinical benefit than other patients. Of 16 patients with strong total Akt2 expression and pAkt Thr308-n+c, 15 (94%) achieved clinical benefit (P=0.077, Fig. 4). Similarly, 15 out of 17 (88%) patients with strong total Akt2 expression and pAkt Ser473-n+c achieved clinical benefit. Even though these results were not statistically significant, there was a clear trend towards clinical benefit in this patient group. These observations, once again, were not reproduced for the Akt1 expression and its combination with pAkt localization.

### Correlation of Akt expression with overall survival

**Figure 5:**
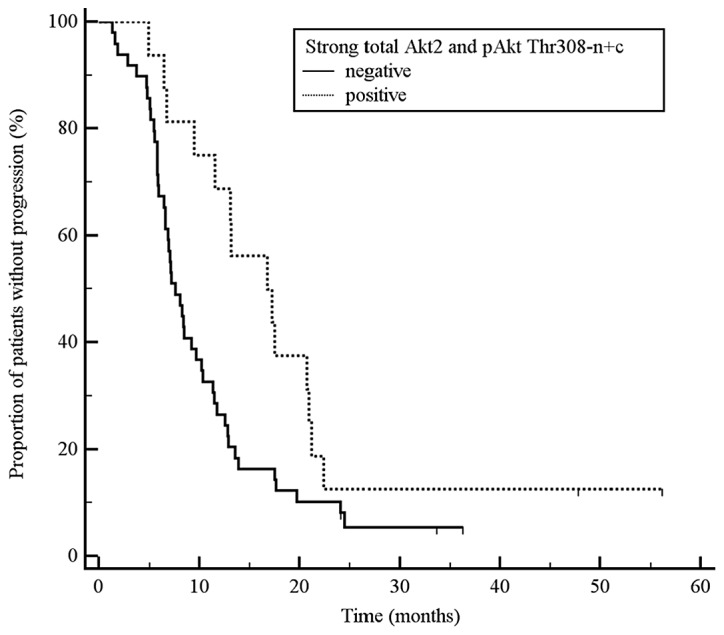
Kaplan-Meier plots of OSt (overall survival from the trastuzumab therapy initiation) for Akt2 expression and concurrent compartmentalization of pAkt Thr308.

For OSt (time from the initiation of trastuzumab therapy for metastatic breast cancer to death from any cause), strong expression of total Akt2 was associated with prolonged survival (40.0 vs. 17.9 months, P=0.03, HR 0.55, 95% CI 0.32–0.93). On the other hand, expression of Akt1, pAkt Thr308 or pAkt Ser473 alone was not associated with better OSt. OSt was longer in patients whose tumours had strong total Akt2 expression and concurrent cytoplasmic and nuclear pAkt activity (strong Akt2 and pAkt Thr308-n+c vs. all others: 51.8 vs. 16.8 months, P=0.0009, HR 0.34, 95% CI 0.19–0.59; strong Akt2 and pAkt Ser473-n+c: 50.8 vs. 17.0 months, P=0.009, HR 0.45, 95% CI 0.26–0.78; Fig. 5).

Similar results were obtained for OSm (time from diagnosis of metastatic breast cancer to death). Strong expression of total Akt2 was associated with prolonged OSm (58.0 vs. 26.4 months, P=0.040, HR 0.57, 95% CI 0.33–0.97). When other markers were analyzed separately, i.e., Akt1, pAkt Thr308 or pAkt Ser473, no correlation was found between these markers and OSm. Analysis of a combination of total Akt expression and compartmentalization of pAkt provide similar results. Strong expression of total Akt2 together with cytoplasmic and nuclear activity of pAkt was associated with longer median OSm. OSm for tumours with strong expression of total Akt2 and pAkt Thr308-n+c vs. all other tumours was 59.2 vs. 24.1 months, P=0.006, HR 0.39, 95% CI 0.22–0.69; and it was 59.2 vs. 24.5 months, P=0.008, HR 0.45, 95% CI 0.26–0.79 for strong expression of total Akt2 and pAkt Ser473-n+c.

### Correlation of Akt expression with disease-free interval

We analyzed the correlation between Akt status and time to disease recurrence in patients who were first diagnosed with early breast cancer and then progressed. We found no association between Akt status (expression of Akt1 and Akt2, and expression and compartmentalization of pAkt Thr308 and pAkt Ser473) and disease free survival, including the analyses of combinations of markers. Neither were there any correlations between Akt expression pattern and the clinical stage of the primary breast cancer at the time of breast cancer diagnosis.

### Univariate and multivariate analyses of TTP, OSt and OSm

The results of univariate Cox regression analyses for TTP, OSt and OSm are summarized in Table III. These results provided the basis for multivariate Cox regression analyses that confirmed independence of some of these parameters in predicting TTP, OSt and OSm (Table III). Strong total expression of Akt2 and concurrent presence of activated pAkt Thr308 in the cytoplasm and nucleus (strong Akt2 and pAkt Thr308-n+c) was an independent positive predictive factor in multivariate analyses for TTP as well as OSt. We consider this as very important, as both these intervals (TTP and OSt) are directly associated with targeted anti-HER2 therapy with trastuzumab. Multivariate analyses also confirmed that the number of metastases is an important negative predictor for all analyzed survival intervals.

## Discussion

In this retrospective study, we investigated associations between Akt expression, activation and compartmentalization and the efficacy of anti-HER2 targeted therapy on a model of metastatic HER2-positive breast cancer patients treated with monoclonal antibody against HER2 receptor, trastuzumab. In this molecular subtype of breast cancers, PI3K/Akt pathway seems to have the highest impact on oncogenic potential of HER2 and the development of resistance to anti-HER2 targeted therapy (8–10).

We confirmed that Akt1 and Akt2 are widely expressed in HER2-positive breast tumours and, simultaneously, tumours contain their activated form. Surprisingly, pAkt did not cross into the nucleus and was found in the cytoplasm only in >20% of tumours (29.7% for pAkt Thr308 and 21.6% for pAkt Ser473). The reasons for this are not known. Nevertheless, biological impact of different compartmentalization of pAkt has been previously shown (29–34). We found that patients with HER2-positive breast cancer treated with anti-HER2 targeted therapy with trastuzumab, whose tumours strongly expressed Akt2, had significantly longer overall survival from targeted treatment initiation (OSt). Furthermore, we found that this anti-HER2 targeted treatment-associated positive effect was even more powerful in patients whose tumours also had strong expression of Akt2 as well as cytoplasmic and nuclear localization of pAkt (pAkt Thr308 and/or pAkt Ser473). These patients had prolonged time to progression, overall survival and more likely achieved clinical benefit (complete or partial remission or disease stabilization). Multivariate analysis confirmed that strong Akt2 expression and pAkt Thr308 (n+c) is a positive and independent predictor of TTP and OSt.

Positive predictive value of Akt2 expression for the outcome of targeted antitumour therapy has been previously described. On a sample of 402 ER-α positive breast cancer patients, Kirkergaard *et al* showed significantly longer survival in a group with tumours presenting strong cytoplasmic expression of Akt2 (HR 1.8, CI 95% 1.14–2.97, P=0.0115) (23). There are two features common to our and Kirgergaard *et al* patient samples. First, all patients were treated with targeted therapy, i.e., a therapy that affects Akt signalling pathway (35). We used anti-HER2 therapy with trastuzumab, Kirgergaard *et al* used anti-ER endocrine therapy with tamoxifen. Second, Akt expression was determined on primary, i.e., treatment naïve tumours. Therefore, we may hypothesize that the reduction of Akt signalling pathway activity by targeted treatment may be associated with better treatment outcome. Similarly to our results, Kirgergaard *et al* did not confirm an association between Akt1 and survival parameters. This may be due to different biological effects of Akt1 and Akt2 in HER2-positive breast cancer (19,20,36,37).

For many years, the oncogenic potential of Akt was considered to originate from its cytoplasmic localization, possibly through regulation of apoptosis, proliferation, energy metabolism and motility, by phosphorylating downstream effectors such as Bad, RAF, CREB, NF-κB, caspases, GSK-3 α/β, mTOR, p21^WAF1^ and others (11,38). However, several studies have identified a pool of activated Akt inside the nucleus (nuclear pAkt). The presence of pAkt in the nucleus mostly results from translocation of pAkt from the cytoplasm (39), although phosphorylation of Akt directly in the nucleus has also been shown, achieved through activated PDK1 that also has the capacity to pass into the nucleus (40). Nuclear Akt has an impact on different biological functions. It has been shown that nuclear Akt regulates cell cycle and apoptosis through phosphorylation of the Forkhead transcription factor (29), GSK-3 (30) and Ebp1 (31). Moreover, nuclear pAkt phosphorylates both cyclin-dependent kinase inhibitors p21^WAF1^ and p27^KIP1^, resulting in their expulsion from the nucleus and subsequent cytoplasmic degradation, thus preventing cell cycle arrest (32–34).

The research studies discussed below provide explanations for the observed impact of pAkt compartmentalization (through direct effect on PI3K/Akt signalling pathway mediated, in our case, by HER2 receptor) on treatment outcome.

Yoo *et al* showed that stimulation of HER3 receptor with its natural ligand heregulin leads to activation and dissociation of Ebp1, a ubiquitously expressed protein, from HER3 and its translocation from the cytoplasm into the nucleus (41). Ahn *et al* confirmed that phosphorylated Ebp1 binds to phosphorylated nuclear Akt and the resulting complex interacts with CAD and inhibits its DNA fragmentation activity; this leads to suppression of apoptosis in the final stage. Moreover, Ebp1 also suppresses caspase-3 substrate ICAD apoptotic degradation (31). These findings suggest that Ebp1 is involved in inhibiting apoptosis on multiple levels. HER3 receptor is then the most frequent heterodimerization partner for HER2 receptor in HER2-positive breast cancer and this dimerization pair forms the most active kinase domain and the strongest PI3K/Akt signalling pathway stimulator (9). Trastuzumab blocks this stimulation. Boehme *et al* showed, that downregulation of Akt2, but not Akt1, by siRNA prevented phosphorylation of GSK-3 and strongly reduced the accumulation of p53 after ionizing irradiation (IR). IR activated predominantly nuclear Akt in a DNA-PK-dependent manner. Nuclear pAkt phosphorylates and thus inactivates GSK-3 very efficiently. Subsequently to inactivation of GSK-3, MDM2 was hypophosphorylated and it was incapable of mediating p53 degradation. In consequence, p53 was accumulated in the nucleus and ready to exert its biological function (30).

Our results and the studies discussed above allow us to hypothesise why patients with HER2-positive breast cancers treated with targeted anti-HER2 therapy achieve better treatment results if their primary tumours have high Akt2 expression and, simultaneously, nuclear pAkt. Constitutive activation of HER2 prior to targeted treatment initiation leads to increased activation of Akt and, through its dimerization partners, HER3 and HER4, also to activation of Ebp1. Activated Akt2 exerts its antiapoptotic and proliferative effects in the cytoplasm. In addition, both phosphorylated molecules, pAkt a pEbp1, cross into the nucleus where they further potentiate these effects. Nuclear pAkt also facilitates stabilization of p53 and its accumulation in the nucleus. Inhibition of PI3K/Akt signalling pathway, with targeted anti-HER2 receptor anticancer treatment in our case, reduces antiapoptotic and proproliferative activity of Akt kinase. On the other hand, in the nuclei of cells with accumulated pAkt and protein p53 leads to cell cycle arrest and subsequent apoptosis. Moreover, lack of pAkt in the nucleus leads to nucleic accumulation of cyclin-dependent kinase inhibitors p21^WAF1^ and p27^KIP1^, resulting in cell cycle arrest (32–34). This hypothesis is supported by the fact that our observations were valid for the survival intervals associated with trastuzumab anti-HER2 therapy (TTP, OSt and OSm) only, not the disease-free survival (DFS). In patients with HER2-positive cancer, DFS depends on adjuvant treatment that, in our sample, did not contain trastuzumab. We found only one study that correlated specifically to nuclear location of Akt with clinical outcome and involved ER-positive breast tumours. Badve *et al* showed that in ER-positive tumours treated with targeted hormonal therapy (circumstances analogous to our study), nuclear location of pAkt was associated with better prognosis (26).

To provide the full picture in this discussion, it should be mentioned that several studies described reverse relationship between Akt and response to different therapy modalities and clinical outcome in breast cancer patients. Activation of Akt was associated with shortened disease-free survival (16,17,21,23,24,28) or overall survival in breast cancer (22). However, cell compartmentalization of pAkt was either not reflected at all in these studies or evidence of pAkt in the cytoplasm was considered as a positive result. In addition, these studies analysed the relationship between Akt and DFS and, with respect to these particular findings, our results do not contravene those of other authors; we did not confirm positive predictive value of strong total Akt2 expression and concurrent pAkt (n+c) on DFS.

No study has been published so far evaluating a relationship between total Akt expression and concurrent subcellular localization of pAkt in primary tumours and the outcome of anti-HER2 targeted therapy. Considering certain inconsistencies in studies on the significance of Akt as well as potential limitations of our study (relatively small numbers of patients, different chemotherapy regimens administered with trastuzumab, different order in which trastuzumab is added to treatment) we suggest that further studies evaluating relationships between Akt, its expression and compartmentalization and the outcome of anticancer treatment affecting the Akt signalling pathway, including *in vitro* studies on cell lines, are conducted before firm conclusions can be made.

In conclusion, even though important advances have been made in the treatment of HER2-positive breast cancer, there is an important group of patients that does not benefit from anti-HER2 targeted therapy as expected. We focused on PI3K/Akt pathway that appears to have the most pronounced impact on oncogenic potential of HER2 and development of resistance to anti-HER2 targeted therapy. We are the first to show the significance of Akt kinase isoform, activity and compartmentalization for prediction of response to trastuzumab-based anti HER2 targeted therapy in patients with HER2-positive metastatic breast cancer; we found that strong Akt2 expression and concurrent presence of activated pAkt in the cytoplasm and nucleus was linked to better outcome. From the confirmed differences in biological function of the various Akt kinase isoforms and the importance of nuclear presence of activated pAkt, we hypothesised why these patients in particular benefited from anticancer treatment that targets the Akt signalling pathway.

## Figures and Tables

**Table I:** Patient and tumour characteristics.

Characteristic	Patients no.	%
Age (years): median 54 (range 32–74)		
<60	57	77.0
≥60	17	23.0
Performance status		
0	25	33.7
1	39	52.7
2	5	6.8
NA	5	6.8
Histology		
Ductal	59	79.7
Lobular	7	9.5
Mixed	2	2.7
Other	6	8.1
Tumour grade		
G1	1	1.3
G2	13	17.6
G3	44	59.5
UN	16	21.6
ER status		
Positive	32	43.2
Negative	40	54.1
NA	2	2.7
PgR status		
Positive	20	27.0
Negative	49	66.2
NA	5	6.8
Position of trastuzumab in palliative therapy		
1. line	44	59.5
2. line	23	31.1
≥3. line	7	9.4
Combination of trastuzumab with cytostatics		
Paclitaxel	39	52.7
Docetaxel	18	24.3
Vinorelbine	8	10.8
CBDCA + paclitaxel	6	8.1
No cytostatics (trastuzumab monotherapy)	3	4.1
Best response to therapy		
CR	9	12.2
PR	33	44.6
SD	19	25.6
PD	8	10.8
NA	5	6.8

CBDCA, carboplatin; CR, complete remission; ER, estrogen receptor; NA, not assessed; no., number; PD, progressive disease; PgR, progesterone receptor; PR, partial remission; SD, stable disease;. UN, unascertained (including a group of tumours where grading was G2–3). All tumours were IHC 3+ or/and FISH-positive.

**Table II:** Akt isoforms expression and compartmentalization in a group of patients with HER2-positive metastatic breast cancer.

Akt isoform	IHC staining	No. of patients	%
Akt1	Negative	17	23.0
	Weak	46	62.1
	Strong	9	12.2
	NA	2	2.7
Akt2	Negative	0	
	Weak	45	60.8
	Strong	26	35.1
	NA	3	4.1
pAkt Thr308	Negative	10	13.5
	Cytoplasmic only	22	29.7
	Nuclear and cytoplasmic	38	51.4
	NA	4	5.4
pAkt Ser473	Negative	5	6.8
	Cytoplasmic only	16	21.6
	Nuclear and cytoplasmic	49	66.2
	NA	4	5.4

NA, not assessed; no., number.

**Table III:** Relationship between studied clinical and molecular factors and survival intervals.

	TTP		OSt		OSm	
Factors	P-value^a^	HR-95% CI^a^	P-value^a^	HR-95% CI^a^	P-value^a^	HR-95% CI^a^
Akt1	0.263	0.411–1.272	0.131	0.334–1.150	0.408	0.417–1.425
Akt2	0.136	0.406–1.128	**0.033**	0.288–0.946	**0.044**	0.292–0.980
pAkt Thr308	0.391	0.797–1.222	0.223	0.541–1.152	0.179	0.536–1.122
pAkt Ser473	0.229	0.488–1.186	0.162	0.459–1.137	0.155	0.464–1.127
Strong Akt2 + pAkt Thr308-n+c	**0.027^b^**	0.276–0.921	**0.002^b^**	0.131–0.620	**0.008**	0.179–0.772
Strong Akt2 + pAkt Ser473-n+c	0.088	0.350–1.072	**0.011**	0.205–0.808	**0.011**	0.196–0.803
ER	0.637	0.685–1.858	0.121	0.365–1.122	**0.014^b^**	0.270–0.861
PgR	0.308	0.769–2.308	0.301	0.757–2.477	0.387	0.718–2.364
Age (<60 vs. ≥60 years)	0.162	0.359–1.184	**0.026**	0.197–0.899	0.056	0.324–1.011
Position of trastuzumab therapy	0.194	0.860–1.840	0.385	0.793–1.832	0.293	0.553–1.194
Number of metastatic sites	**0.004^b^**	**1.114–1.751**	**0.002^b^**	**1.164–1.947**	**0.014^b^**	**1.070–1.797**

HR-95% CI, 95% confidence interval for the hazard ratio; ER, estrogen receptor; PgR, progesterone receptor; OSt, overall survival as a time from the initiation of trastuzumab based treatment to death from any cause; OSm, overall survival as a time from diagnosis of metastatic disease to death from any cause; TTP, time from the initiation of trastuzumab based treatment to the disease progression.

a Results of the univariate Cox regression analysis;

b multivariate Cox regression analysis confirmed independence of this predictive factor (P<0.05).

## References

[1] Slamon DJ, Clark GM, Wong SG, Levin WJ, Ullrich A, McGuire WL (1987). Human breast cancer: correlation of relapse and survival with amplification of the HER-2/neu oncogene. Science.

[2] Owens MA, Horten BC, Da Silva MM (2004). HER2 amplification ratios by fluorescence in situ hybridization and correlation with immunohistochemistry in a cohort of 6556 breast cancer tissues. Clin Breast Cancer.

[3] Cobleigh MA, Vogel CL, Tripathy D, Robert NJ, Scholl S, Fehrenbacher L, Wolter JM, Paton V, Shak S, Lieberman G, Slamon DJ (1999). Multinational study of the efficacy and safety of humanized anti-HER2 monoclonal antibody in women who have HER2-overexpressing metastatic breast cancer that has progressed after chemotherapy for metastatic disease. J Clin Oncol.

[4] Baselga J, Tripathy D, Mendelsohn J, Baughman S, Benz CC, Dantis L, Sklarin NT, Seidman AD, Hudis CA, Moore J (1996). Phase II study of weekly intravenous recombinant humanized anti-p185^HER2^ monoclonal antibody in patients with HER2/neu-overexpressing metastatic breast cancer. J Clin Oncol.

[5] Vogel CL, Cobleigh MA, Tripathy D, Gutheil JC, Harris LN, Fehrenbacher L, Slamon DJ, Murphy M, Novotny WF, Burchmore M (2002). Efficacy and safety of trastuzumab as a single agent in first-line treatment of HER2-overexpressing metastatic breast cancer. J Clin Oncol.

[6] Slamon DJ, Leyland-Jones B, Shak S, Fuchs H, Paton V, Bajamonde A, Fleming T, Eiermann W, Wolter J, Pegram M (2001). Use of chemotherapy plus a monoclonal antibody against HER2 for metastatic breast cancer that overexpresses HER2. N Engl J Med.

[7] Yin W, Jiang Y, Shen Z, Shao Z, Lu J (2011). Trastuzumab in the adjuvant treatment of HER2-positive early breast cancer patients: a meta-analysis of published randomized controlled trials. PLoS One.

[8] Arteaga CL, Sliwkowski MX, Osborne CK, Perez EA, Puglisi F, Gianni L (2012). Treatment of HER2-positive breast cancer: current status and future perspectives. Nat Rev Clin Oncol.

[9] Hsieh AC, Moasser MM (2007). Targeting HER proteins in cancer therapy and the role of the non-target HER3. Br J Cancer.

[10] She QB, Chandarlapaty S, Ye Q, Lobo J, Haskell KM, Leander KR, DeFeo-Jones D, Huber HE, Rosen N (2008). Breast tumor cells with PI3K mutation or HER2 amplification are selectively addicted to Akt signaling. PLoS One.

[11] Vivanco I, Sawyers CL (2002). The phosphatidylinositol 3-Kinase AKT pathway in human cancer. Nat Rev Cancer.

[12] Sarbassov DD, Guertin DA, Ali SM, Sabatini DM (2005). Phosphorylation and regulation of Akt/PKB by the rictor-mTOR complex. Science.

[13] Bozulic L, Surucu B, Hynx D, Hemmings BA (2008). PKBalpha/Akt1 acts downstream of DNA-PK in the DNA double-strand break response and promotes survival. Mol Cell.

[14] Bacus SS, Altomare DA, Lyass L, Chin DM, Farrell MP, Gurova K, Gudkov A, Testa JR (2002). AKT2 is frequently upregulated in HER-2/neu-positive breast cancers and may contribute to tumor aggressiveness by enhancing cell survival. Oncogene.

[15] Park SS, Kim SW (2007). Activated Akt signaling pathway in invasive ductal carcinoma of the breast: correlation with HER2 overexpression. Oncol Rep.

[16] Zhou X, Tan M, Stone Hawthorne V, Klos KS, Lan KH, Yang Y, Yang W, Smith TL, Shi D, Yu D (2004). Activation of the Akt/mammalian target of rapamycin/4E-BP1 pathway by ErbB2 overexpression predicts tumor progression in breast cancers. Clin Cancer Res.

[17] Tokunaga E, Kimura Y, Oki E, Ueda N, Futatsugi M, Mashino K, Yamamoto M, Ikebe M, Kakeji Y, Baba H, Maehara Y (2006). Akt is frequently activated in HER2/neu-positive breast cancers and associated with poor prognosis among hormone-treated patients. Int J Cancer.

[18] Stål O, Pérez-Tenorio G, Akerberg L, Olsson B, Nordenskjöld B, Skoog L, Rutqvist LE (2003). Akt kinases in breast cancer and the results of adjuvant therapy. Breast Cancer Res.

[19] Hutchinson JN, Jin J, Cardiff RD, Woodgett JR, Muller WJ (2004). Activation of Akt-1 (PKB-alpha) can accelerate ErbB-2-mediated mammary tumorigenesis but suppresses tumor invasion. Cancer Res.

[20] Arboleda MJ, Lyons JF, Kabbinavar FF, Bray MR, Snow BE, Ayala R, Danino M, Karlan BY, Slamon DJ (2003). Overexpression of AKT2/protein kinase Bbeta leads to up-regulation of beta1 integrins, increased invasion, and metastasis of human breast and ovarian cancer cells. Cancer Res.

[21] Pérez-Tenorio G, Stål O (2002). Activation of AKT/PKB in breast cancer predicts a worse outcome among endocrine treated patients. Br J Cancer.

[22] Schmitz KJ, Otterbach F, Callies R, Levkau B, Hölscher M, Hoffmann O, Grabellus F, Kimmig R, Schmid KW, Baba HA (2004). Prognostic relevance of activated Akt kinase in node-negative breast cancer: a clinicopathological study of 99 cases. Mod Pathol.

[23] Kirkegaard T, Witton CJ, McGlynn LM, Tovey SM, Dunne B, Lyon A, Bartlett JM (2005). AKT activation predicts outcome in breast cancer patients treated with tamoxifen. J Pathol.

[24] Vestey SB, Sen C, Calder CJ, Perks CM, Pignatelli M, Winters ZE (2005). Activated Akt expression in breast cancer: correlation with p53, Hdm2 and patient outcome. Eur J Cancer.

[25] Andre F, Nahta R, Conforti R, Boulet T, Aziz M, Yuan LX, Meslin F, Spielmann M, Tomasic G, Pusztai L (2008). Expression patterns and predictive value of phosphorylated AKT in early-stage breast cancer. Ann Oncol.

[26] Badve S, Collins NR, Bhat-Nakshatri P, Turbin D, Leung S, Thorat M, Dunn SE, Geistlinger TR, Carroll JS, Brown M (2010). Subcellular localization of activated AKT in estrogen receptor-and progesterone receptor-expressing breast cancers: potential clinical implications. Am J Pathol.

[27] Esteva FJ, Guo H, Zhang S, Santa-Maria C, Stone S, Lanchbury JS, Sahin AA, Hortobagyi GN, Yu D (2010). PTEN, PIK3CA, p-AKT, and p-p70S6K status: association with trastuzumab response and survival in patients with HER2-positive metastatic breast cancer. Am J Pathol.

[28] Wu Y, Mohamed H, Chillar R, Ali I, Clayton S, Slamon D, Vadgama JV (2008). Clinical significance of Akt and HER2/neu overexpression in African-American and Latina women with breast cancer. Breast Cancer Res.

[29] Brunet A, Bonni A, Zigmond MJ, Lin MZ, Juo P, Hu LS, Anderson MJ, Arden KC, Blenis J, Greenberg ME (1999). Akt promotes cell survival by phosphorylating and inhibiting a Forkhead transcription factor. Cell.

[30] Boehme KA, Kulikov R, Blattner C (2008). p53 stabilization in response to DNA damage requires Akt/PKB and DNA-PK. Proc Natl Acad Sci USA.

[31] Ahn JY, Liu X, Liu Z, Pereira L, Cheng D, Peng J, Wade PA, Hamburger AW, Ye K (2006). Nuclear Akt associates with PKC-phosphorylated Ebp1, preventing DNA fragmentation by inhibition of caspase-activated DNase. EMBO J.

[32] Zhou BP, Liao Y, Xia W, Spohn B, Lee MH, Hung MC (2001). Cytoplasmic localization of p21^Cip1/WAF1^ by Akt-induced phosphorylation in HER-2/neu-overexpressing cells. Nat Cell Biol.

[33] Viglietto G, Motti ML, Bruni P, Melillo RM, D’Alessio A, Califano D, Vinci F, Chiappetta G, Tsichlis P, Bellacosa A (2002). Cytoplasmic relocalization and inhibition of the cyclin-dependent kinase inhibitor p27^Kip1^ by PKB/Akt-mediated phosphorylation in breast cancer. Nat Med.

[34] Le XF, Pruefer F, Bast RC (2005). HER2-targeting antibodies modulate the cyclin-dependent kinase inhibitor p27^Kip1^ via multiple signaling pathways. Cell Cycle.

[35] Simoncini T, Hafezi-Moghadam A, Brazil DP, Ley K, Chin WW, Liao JK (2000). Interaction of oestrogen receptor with the regulatory subunit of phosphatidylinositol-3-OH kinase. Nature.

[36] Irie HY, Pearline RV, Grueneberg D, Hsia M, Ravichandran P, Kothari N, Natesan S, Brugge JS (2005). Distinct roles of Akt1 and Akt2 in regulating cell migration and epithelial-mesenchymal transition. J Cell Biol.

[37] Yoeli-Lerner M, Yiu GK, Rabinovitz I, Erhardt P, Jauliac S, Toker A (2005). Akt blocks breast cancer cell motility and invasion through the transcription factor NFAT. Mol Cell.

[38] Datta SR, Brunet A, Greenberg ME (1999). Cellular survival: a play in three Akts. Genes Dev.

[39] Xuan Nguyen TL, Choi JW, Lee SB, Ye K, Woo SD, Lee KH, Ahn JY (2006). Akt phosphorylation is essential for nuclear translocation and retention in NGF-stimulated PC12 cells. Biochem Biophys Res Commun.

[40] Kikani CK, Dong LQ, Liu F (2005). ‘New’-clear functions of PDK1: beyond a master kinase?. J Cell Biochem.

[41] Yoo JY, Wang XW, Rishi AK, Lessor T, Xia XM, Gustafson TA, Hamburger AW (2000). Interaction of the PA2G4 (EBP1) protein with ErbB-3 and regulation of this binding by heregulin. Br J Cancer.

